# Detection in whole blood of autoantibodies for the diagnosis of connective tissue diseases in near patient testing condition

**DOI:** 10.1371/journal.pone.0202736

**Published:** 2018-08-30

**Authors:** Makoto Miyara, Jean-Luc Charuel, Sasi Mudumba, Alice Wu, Pascale Ghillani-Dalbin, Zahir Amoura, Rufus W. Burlingame, Lucile Musset

**Affiliations:** 1 APHP, Immunology department, Pitié-Salpétrière Hospital, Paris, France; 2 Sorbonne Université, INSERM U1135, Centre d’Immunologie et des Maladies Infectieuses (CIMI), Paris, France; 3 Genalyte Inc., San Diego, CA, United States of America; 4 APHP, Service de Médecine Interne 2, institut E3M, Centre de référence national du lupus et du syndrome des antiphospholipides,Hôpital Pitié-Salpêtrière Hospital, Paris, France; University of Bergen, NORWAY

## Abstract

A novel technology, photonic ring immunoassay (PRI), for detecting 12 autoantibodies simultaneously in whole blood in less than 15 minutes was evaluated by comparing results from 235 clinically diagnosed patients with standard laboratory tests. The overall agreement was greater than 91% for 10 of the 12 assays, with positive percent agreement greater than 89% for 9 of the assays and negative percent agreement greater than 91% for 10 of them. Thus, the clinical sensitivities and specificities were similar for the 2 methods. In addition, 199 normal blood donors were tested on the ANA 12 PRI, yielding specificities greater than 97.5% for all assays. This proof of concept study shows that this new system is suitable for point of care testing for clinically useful autoantibodies, allowing the doctor to have test results in minutes rather than days.

## Introduction

There has recently been discussion that point of care testing for markers of autoimmune rheumatic diseases, with results reported in real time, would be beneficial to patients [1–4]. The algorithm to search for autoantibodies to extractable nuclear antigens (anti-ENA) in patients showing clinical symptoms of a connective tissue disease (CTD) usually starts with the test for anti-nuclear antibodies (ANA) by indirect immunofluorescence on HEp-2 cells[2,5,6]. When a positive fluorescence pattern is observed with a given serum, it is necessary to determine the specificity of those autoantibodies by determining the autoantigens that are bound to them using any of several commercially available single-plex tests like ELISAs, or multiplex systems such as bead immunoassays or immunoblotting, or both. This follow up testing is necessary since a positive ANA can be caused by many autoantibodies that are not clinically relevant [7], as well as certain autoantibodies that are strongly correlated with specific autoimmune diseases[6,8,9] including some that are part of the diagnostic criteria[10–13]. In rare cases samples that are negative for immunofluorescence on HEp-2 cells are positive on follow up tests [14,15]. Anti-SS-A and anti-SS-B are related to Sjögren’s syndrome[10]; anti-dsDNA, anti-nucleosome, anti-Sm, anti-Ribosome P and anti-PCNA autoantibodies are associated with systemic lupus erythematosus (SLE)[11,16,17]; anti-RNP autoantibodies are correlated with mixed connective tissue disease[12]; anti-centromere, anti-Scl-70 and anti-Ku are associated with systemic sclerosis, and anti-Jo-1 autoantibodies are found in myositis [13,18,19]. Anti-SS-A and anti-RNP are also associated with SLE.

Most single-plex autoantibody assays are designed to be performed in a batch on a serum sample. Thus, the drawn blood has to be transported to a lab where it is centrifuged to separate serum from the blood, the serum is aliquoted into separate tubes for each test and then run on the separate tests. These steps lead to delays of a day or longer in the delivery of results to the physician and the patients.

Genalyte has developed a novel multiplex detection technology based on flowing diluted whole blood over a silicon chip that yields results in 15 minutes. This allows the doctor to receive results while the patient is still in the office or clinic. The technique uses photonic ring resonance to measure binding of macromolecules to sensors on a miniature silicon chip[20–22]. The Maverick Detection System detects changes in resonance wavelength as macromolecules such as antibodies, other proteins or nucleic acids bind to the sensors. The technology is therefore suitable for the detection of autoantibodies when specific antigens are bound to the sensors.

Here we show in a proof of concept study that the use of a photonic ring immunoassay (PRI) enables the detection of anti-ENA in whole blood within a short period leading to rapid diagnostic test results in near patient or point of care testing conditions. Comparisons of the results from the PRI are made to both the results of standard laboratory tests and the clinical diagnosis of the patients.

## Methods

### Patients and samples

Routinely drawn whole blood from 235 consecutive patients followed-up between March and June 2016 at the Pitié-Salpétrière hospital (Paris, France) was analyzed in the clinical lab. One aliquot of blood was processed into serum for testing with the routine laboratory procedures as described below, and a paired whole blood sample and serum sample were both tested on the ANA 12 PRI. One hundred forty two patients had systemic lupus erythematosus (SLE), 13 had Sjögren's syndrome, 10 had primary anti-phospholipid syndrome, 6 had ANCA associated vasculitis, 4 had Raynaud’s phenomenon, 4 had rheumatoid arthritis, 5 had systemic sclerosis and 3 had myositis. Forty eight other patients had a final diagnosis different from CTD. CTD diagnosis were made by the clinicians. All patients fulfilled the international consensus criteria for those diseases [10–13]. All 235 samples were tested on the ANA 12 PRI, while some samples were not tested for all 12 analytes with the routine laboratory procedures because of the algorithm for testing used in the lab.

One strength of the above patient population is that both the serologic evaluation and the diagnosis are known. Most of the patients were diagnosed with a CTD, predominately SLE, which is the population the ANA 12 PRI tests were designed to help diagnose. However, this population limits the ability to test for the specificity of the new technology in a non-disease population. To better evaluate clinical specificity of the ANA 12 PRI, 199 serum samples from presumptively normal donors were purchased from serum vendors (PromedX, Bioreclamation) and tested in the research laboratory at Genalyte, Inc. We should note that antibodies to help diagnose ANCA associated vasculitis, such as MPO and PR3, and rheumatoid arthritis, like RF and CCP, were not tested.

Because this was a method comparison study with blinded samples for i*n vitro* diagnostic testing for widely accepted biological parameters, this non-interventional study did not require review by a local ethical board or formal written consent.

### Routine laboratory autoantibody testing procedures

For a given sample, the search for anti-nuclear antibodies aims at detecting diagnostically important autoantibodies recognizing autoantigens residing either in the nucleus or the cytoplasm or both of HEp-2 cells to aid in the diagnosis of CTD[2,3,6,8,9,14,15]. To this end, indirect immunofluorescence is performed first on HEp-2 cells. When positive, the tests to detect specific antibodies were performed through a multiplex ENA assay on the FIDIS instrument (Theradiag, France), anti-DNA ELISA (DiaSorin), Farr assay (Trinity Biotech) and/or anti-nucleosome ELISA (Werfen). Discrepancies between the findings made by immunofluorescence and specific tests mentioned above can occur. For example, a sample showing a course speckled immunofluorescent pattern on HEp-2 cells that is consistent with the presence of anti-Sm or anti-RNP autoantibodies might be negative for these autoantibodies on the FIDIS ENA assay. In this case, immunoblotting tests (D-tek) are performed to draw a definite conclusion about the autoantibody reactivity in that sample. In addition, anti-dsDNA is evaluated on the FIDIS, ELISA and Farr assays. The algorithm in the lab to determine reactivity to dsDNA considers the results form the Farr assay as the gold standard. Thus, the final conclusion concerning the autoantibody profile of a given patient may employ several immunological techniques. In this algorithm not every sample is tested for every autoantibody. For example, a sample with a speckled ANA pattern is not necessarily tested for anti-dsDNA antibodies.

### ANA 12 photonic ring immunoassay

The ANA 12 Photonic Ring Immunoassay (PRI, Genalyte Inc.) has been designed to enable the detection of IgG autoantibodies recognizing SS-A 60, SS-B, Sm, RNP, Ribosome P, PCNA, Scl-70, Centromere protein B, Ku, Jo-1, dsDNA and nucleosome in a single multiplexed immunoassay using whole blood or serum. The ANA 12 PRI assay is based on the Maverick™ Detection System (Genalyte, Inc. USA) which performs multiplexed detection of autoantibody binding events by measuring the shift in wavelength of ring resonance as the antibodies bind to the antigens on the surface above the rings[20,22].

For each sample, a small aliquot of whole blood (10μl) or serum (5μl) is mixed with 250μl of diluent in the sample well of the consumable array, which is then placed in the Maverick instrument. The same buffer is used for sample diluent, running buffer, wash and anti-IgG diluent. It is 0.15M phosphate buffered saline with 1% human serum albumin (SeraCare), 0.5% Tween 20 and 0.09% sodium azide. The instrument performs all subsequent steps automatically and results for each sample are obtained in less than 15 minutes. Briefly, the chip is equilibrated with buffer for a minute to get a raw baseline resonance for every ring on the chip. At this stage of the assay all rings are set to zero resonant wavelength. Then the diluted blood or serum is flowed over the chip for 2.5 minutes to allow binding of any specific autoantibodies to the antigens on the chip, followed by a rinse for 1.5 minutes to wash away non-specifically bound molecules and the average of the last 30 seconds of this rinse is the initial baseline for the next step. At this stage of the assay the total of specific IgG and non-specific binding of macromolecules to the antigen above the rings is measured. In the detection step, 0.1 mg/ml of goat anti-human IgG (Jackson Immunoresearch) is flowed over the chip for 3.5 minutes and allowed to bind to any IgG from the patient that is on any of the antigens. A shift in resonant wavelength is observed as the anti-IgG binds to any human IgG that is bound to the antigen. This significantly amplifies the signal from the IgG bound to the antigen. The average of the last 30 seconds is used as the ending baseline. The specific IgG signal is the difference between the initial baseline and the ending baseline.

The change in the resonant wavelength, expressed in Genalyte Response Units (GRU) is proportional to the mass bound above the ring resonators. A specific cutoff in GRU is determined for each antigen for every lot of the ANA 12 chip, and the measured GRU for each autoantibody are divided by the value of the corresponding specific cutoff to yield Arbitrary Units (AU), with <30 AU negative, 30–40 AU indeterminate, and >40 AU positive. Expressing results as a ratio yielding arbitrary units is common in most commercial ELISA and Luminex tests that measure autoantibodies that do not have international standards. Another advantage of this technique is that all cutoffs between positive and negative can be made uniform in AU even when the GRU for the cutoff are different. For all calculations in this paper the indeterminate results were considered to be negative because in the clinical lab all indeterminate results on the FIDIS are considered negative. There were very few samples in the indeterminate range, so the conclusions would not change if the indeterminate samples were excluded or considered positive.

### Method comparison with results from the clinical laboratory

The performance of the ANA 12 PRI assay using whole blood was evaluated by comparing results to those obtained using serum for the routine testing procedures in the lab. All samples were anonymized. True positive and true negative refer to the final conclusion from the standard laboratory tests. False positive and false negative results are those where the ANA 12 is positive when the routine lab result is negative, and when the ANA 12 PRI is negative when the routine lab result is positive, respectively. Positive, negative and total agreements were calculated for each of the autoantibodies as follows:

Positive agreement = True Positive / (True Positive + False Negative);

Negative agreement = True Negative / (True negative + False Positive);

Total agreement = (True positive + True negative) / (True Positive + True Negative + False Positive + False Negative).

In addition to the qualitative comparison, the quantitative results were compared by performing linear regression analysis on the results from the ANA PRI on Maverick compared to FIDIS or Farr assays.

### Diagnostic comparison

The performances of both the routine laboratory procedures and the ANA 12 PRI were also evaluated by comparing results to the clinical diagnosis of the patients:

Sensitivity = Number positive for an antibody/Number with the associated disease.

Specificity = (Number with non-associated disease—Number positive for antibody) / Number with non-associated disease.

### Comparison of whole blood and serum on the maverick

Both qualitative and quantitative analyses were performed comparing results from whole blood to those from serum on the ANA 12 PRI.

## Results

### Comparison of qualitative results of whole blood on ANA 12 PRI to results from the laboratory

The results from the ANA 12 PRI showed excellent total, positive and negative percent agreement when compared to the conclusions drawn from the routine testing procedures in the laboratory (Table 1). For Sm, Scl-70, Jo-1, SS-A 60, and Ku antigens the total, positive and negative percent agreement were all above 93%. PCNA was above 92% and Centromere and SS-B were above 89%. For RNP, total agreement was 91%, positive was 100% and negative was 88%. For Ribosome P, the overall agreement and specificity were greater than 90%, but the sensitivity was lower. The anti-nucleosome and anti-dsDNA results from the ANA 12 PRI displayed lower agreement with the conclusion of the lab than the other antigens. For the detection of anti-DNA antibodies, several techniques were utilized: FIDIS (n = 130), ELISA (n = 60), or Farr (n = 141), because the discrepancies between those techniques to measure anti-dsDNA antibodies are well known[23,24].

**Table 1:** Positive and negative percent agreement with laboratory results.

	Laboratory Conclusion									ELISA		Farr
Conclusion vs PRI	Sm	RNP	SS-A 60	SS-B	Ribo P	PCNA	Scl-70	Centromere B	Jo-1	Nucleosome	Ku	dsDNA
True Positive (++)	23	50	60	16	11	5	3	8	1	38	3	43
False Negative (+-)	0	0	0	2	8	0	0	1	0	8	0	9
False Positive (-+)	14	20	2	7	1	10	0	2	0	13	0	24
True Negative (—)	194	161	169	206	105	113	227	215	229	55	1	65
Positive Agreement	100%	100%	100%	89%	58%	100%	100%	89%	100%	83%	100%	83%
Negative Agreement	93%	89%	99%	97%	99%	92%	100%	99%	100%	81%	100%	73%
Overall Agreement	94%	91%	99%	96%	93%	92%	100%	99%	100%	82%	100%	77%

### Comparison of quantitative results between ANA 12 PRI, Fidis, ELISA and Farr assays

The results from most autoantibody tests, including the ANA 12 PRI, are expressed in semi-quantitative units (AU/mL). A high positive result can give the doctor more confidence that the result is a clinically relevant positive compared to a result just above the cutoff between negative and positive. Linear regression analysis shows that some antigens, such as SS-A 60, SS-B, Ribosome P, Scl-70 and Centromere B have good quantitative correlation with the corresponding results on FIDIS, with r^2^ greater than 0.72 (Table 2). The slope of the line can be greater than 3, showing that even though there is good agreement between high and low positive samples, the absolute values are different. Most of the other assays showed moderate agreement. The anti-dsDNA results on the ANA 12 PRI did not show quantitative agreement with the Farr assay, yielding r^2^ of 0.23. This result was expected given the wide variation shown by different technologies to detect anti-dsDNA.

**Table 2:** Comparison of quantitative values from FIDIS.

Antigen	Slope and intercept	Correlation (r^2^)
Sm	Y = 1.8x+28.2	r^2^ = 0.69
RNP	Y = 3.1x+93.8	r^2^ = 0.55
SS-A 60	Y = 3.2x+2.5	r^2^ = 0.82
SS-B	Y = 2.8x+1.3	r^2^ = 0.76
Ribosome P	Y = 1.4x+1.0	r^2^ = 0.83
dsDNA^*^	Y = 1.8x+36.0	r^2^ = 0.28
Scl-70	Y = 2.2x+2.5	r^2^ = 0.72
Centromere B	Y = 1.7x+6.9	r^2^ = 0.82
Jo-1	Y = 0.7x+8.2	r^2^ = 0.48

Note:

* dsDNA is compared to Farr

### Results of normal blood donors on ANA 12 PRI

The results from testing samples from normal human donors show high levels of specificity for each analyte from 97.0 to 100% (Table 3). Thus, each of the 12 assays is highly specific.

**Table 3:** Clinical specificity of ANA 12 PRI.

N = 199 Antigen	Mean (AU/mL)	Number True Negative	Specificity
Sm	<2	198	99.5%
RNP	9.4	195	98.0%
SS-A 60	6.8	199	100.0%
SS-B	<2	197	99.5%
Ribosome P	4.6	198	99.5%
PCNA	<2	198	99.5%
Scl-70	7.4	198	99.5%
Centromere	6.0	198	99.5%
Jo-1	3.7	199	100.0%
Nucleosome	4.4	199	100.0%
Ku	<2	198	99.5%
dsDNA	3.0	193	97.0%

### Comparison of whole blood to serum on the ANA 12 PRI

**Fig 1:**
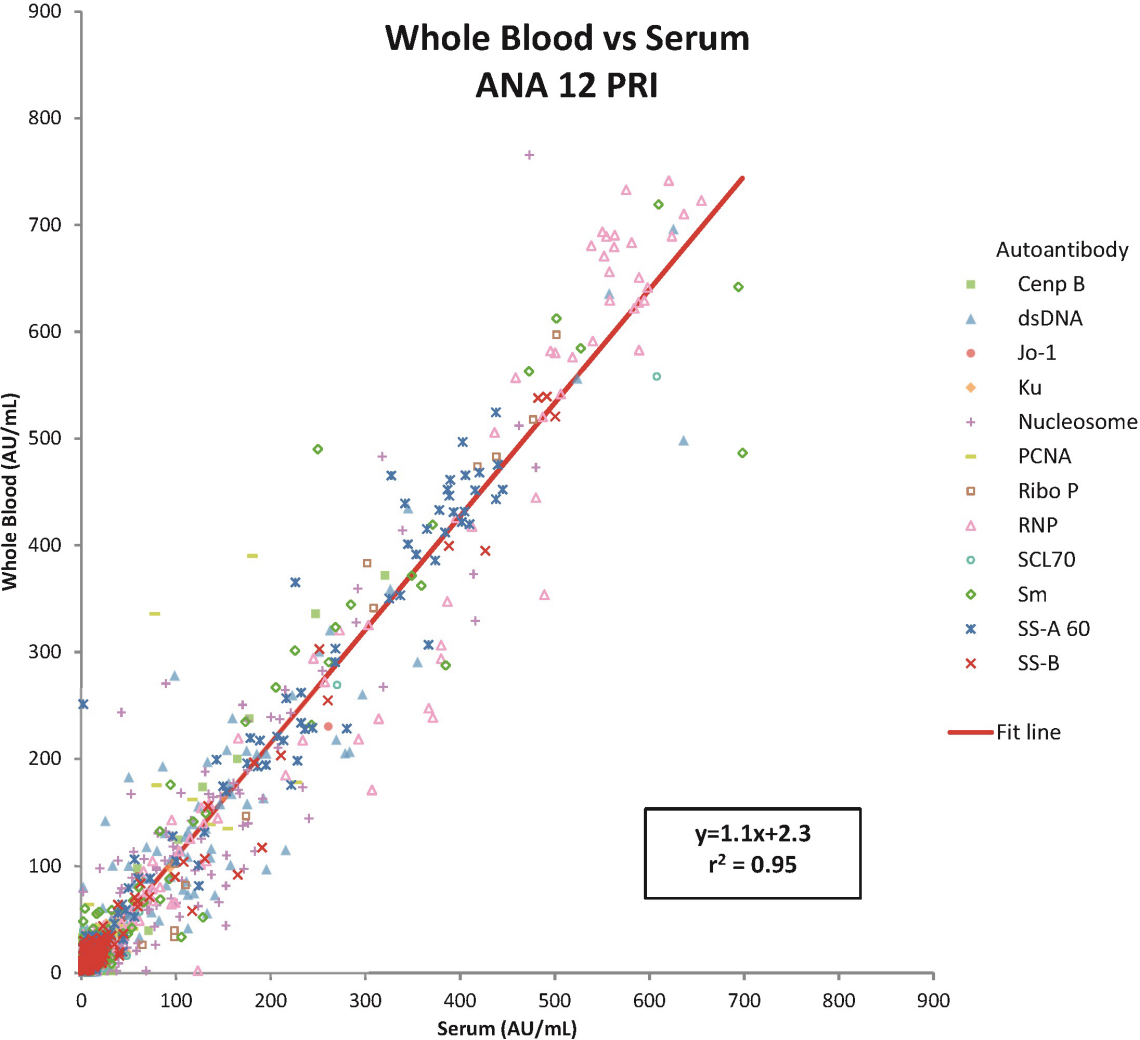
Combined whole blood vs serum correlation.

In terms of positive and negative results, whole blood and serum gave virtually identical results, with agreement between 97%-100% for all tests except Sm and dsDNA at 96% and 95% respectively. Discrepant results were all near the cutoff. The qualitative results were also comparable, yielding correlation coefficients greater than 0.90 for 9 of the antigens (Table 4). Two of the exceptions, PCNA and Ku, had a very small number of positive samples, so noise in the negative samples contributed to the lower r^2^ values. Nucleosome was in between with r^2^ of 0.86. Correlation between whole blood and serum results when all are combined is r^2^ = 0.95 (Fig 1)

**Table 4:** Whole blood vs serum correlations for ANA 12 PRI.

Autoantibody	Equation	R^2^
Sm	Y = 1.03x+5.7	0.94
RNP	Y = +1.1x+1.4	0.97
SS-A 60	Y = 1.1x+4.0	0.97
SS-B	Y = 1.0x+2.0	0.97
Ribosome P	Y = 1.1x+1.2	0.98
PCNA	Y = 1.3x+3.9	0.68
Scl-70	Y = 0.9x+3.3	0.97
Centromere	Y = 1.2x+1.1	0.95
Jo-1	Y = 0.8x+3.6	0.90
Nucleosome	Y = 1.1x+0.67	0.86
Ku	Y = 0.9x+3.0	0.77
dsDNA	Y = 0.99x+3.7	0.90

### Correlation of discrepant results with clinical diagnosis

The results from the routine testing procedures in the lab were shared with the physicians and were used to help diagnose the patients. In contrast, the results from the ANA 12 PRI were never shared with the clinicians. As expected from the good correlation between the general lab results and those from the ANA 12 PRI, the ANA 12 showed the expected clinical sensitivity and specificity (Table 5) compared to the lab results. However, there were a few autoantibodies that showed differences.

**Table 5:** Clinical sensitivity and specificity.

Autoantibody	Disease State	Lab Results		ANA 12 PRI Results	
		Sensitivity	Specificity	Sensitivity	Specificity
Sm	Lupus	16%	100%	25%	99%
RNP	Lupus	34%	98%	48%	97%
SS-A 60	Lupus	36%	90%	37%	90%
dsDNA	Lupus	56%	100%	53%	92%
Nucleosome	Lupus	53%	97%	52%	91%
Ribosome P	Lupus	20%	100%	8%	100%
PCNA	Lupus	0%	100%	10%	100%
SS-B	Sjögren’s/Lupus	12%	100%	15%	100%
Scl-70	Scleroderma	20%	99%	20%	99%
Centromere	Scleroderma	60%	97%	60%	97%
Ku	Myositis/Scleroderma	0%	50%	0%	98%
Jo-1	Myositis	33%	100%	33%	100%

For some autoantibodies, such as Sm, RNP, SS-B and PCNA, the PRI had more positive results than the standard lab tests. When correlating the results obtained with ANA 12 PRI with the clinical diagnosis, we found that 13 of 14 samples that were positive for Sm using the ANA 12 PRI but negative by the lab test were from patients diagnosed with SLE. All 13 of these Sm positive SLE patients were also positive for RNP. Thus, there was higher sensitivity with only 1 clinical false positive for Sm.

The same observation was true for RNP. That is 19 of the 20 samples that were positive for RNP by ANA 12 PRI but negative by the lab conclusion were diagnosed with SLE, and the other sample was from a Sjögren's syndrome patient. It should be pointed out that the immunofluorescent pattern for these samples was consistent with the presence of Sm/RNP autoantibodies. In addition, both of these tests showed greater than 99% specificity when tested on samples from normal blood donors. Only 1 of the 93 patients without SLE in the clinical group was positive for anti-Sm, yielding 99% clinical specificity in this group as well. Thus, these positive results are likely to be caused by the presence of specific Sm and RNP autoantibodies in these patients and not non-specific binding.

There were only 2 discrepant results for anti-SS-A 60 autoantibodies, both positive for ANA 12 PRI but negative by lab tests, and both had lupus. Similar results where the ANA 12 PRI was positive but the conclusion of the lab was negative were found for SS-B, Ribosome P and PCNA where all 7, 1 and 10 discrepant samples, respectively, had lupus. Since these autoantibodies are all associated with lupus, the added sensitivity could be useful. On the other hand there were 3 lupus patients where the ANA 12 PRI was positive for Centromere B and the lab results were negative. These would be considered clinical false positive results since anti-Centromere B are associated with scleroderma. However the presence of anti-Centromere B has already been described in 1.9% of patients with SLE, indicating that the Maverick system has the same sensitivity for anti-Centromere B in SLE as the systems used in a previous study[25].

For Ribosome P the PRI had less positive results than the laboratory test. However, all cases with false negative results for Ribosome P with the ANA 12 PRI showed positive results for other specific autoantibodies associated with SLE. Therefore, no diagnosis of SLE would have been missed by using the ANA PRI 12. There were no discrepant results between the ANA PRI 12 and the conclusion of the lab for Jo-1, Scl-70 and Ku.

As expected when anti-dsDNA tests are performed on different technologies, there were more discrepant results than found in the other tests [23,24]. The conclusion of many studies performed in the 1990s was that the different types of dsDNA assays presented different epitopes on DNA for binding, and many people have an anti-DNA response that is restricted to a limited number of epitopes. Thus, the fact that the dsDNA results are the most different between the lab and ANA 12 PRI was expected. All 10 samples that were positive for the conclusion of the lab but negative on ANA 12 PRI had SLE, while 19 of the 23 samples that were positive on ANA 12 PRI but negative by the conclusion of the lab had SLE. Since there are 93 patients in this group who do not have SLE, the clinical specificity of the dsDNA PRI is 96%.

If we only compare the 141 samples tested by the Farr assay, 43 were positive on both, 65 negative on both, 9 positive on Farr but negative by PRI, and 24 positive by PRI but negative by Farr (Table 1).

For nucleosome, all 8 samples positive on the lab conclusion but negative on the ANA 12 PRI had lupus, and 9 of 13 that were positive on the ANA 12 PRI but negative by lab conclusion had lupus. The other 4 had Sjögren's syndrome. Thus, the overall clinical sensitivities and specificities for anti-dsDNA and anti-nucleosome were similar for the PRI and the lab tests.

## Discussion

In this study, we have demonstrated that the ANA 12 PRI was efficient in detecting diagnostically important anti-nuclear antibodies in whole blood. There was excellent correlation with the predicate devices used in the lab, and the clinical sensitivity and specificity were as expected. The contribution of the ANA 12 PRI to the global diagnosis of CTD, either positive or negative, was as good as the global routine laboratory procedures for specific autoantibodies. The obvious difference between the use of the ANA 12 PRI assays and the use of the whole routine laboratory algorithm is the speed in obtaining the results. The ANA 12 PRI enables the delivery of the results within 15 minutes from drawing the blood. Therefore, the system is suitable for point of care testing in rheumatology clinics or doctors’ offices. More generally, this technology could be useful for clinical settings requiring rapid results i.e. near patient diagnosis such as critical care situations.

Because most symptoms associated with CTD such as joint pain, skin rash, or fatigue are poorly specific for any disease, it is necessary to perform diagnostic tests to rule out or rule in the diagnosis of CTD. Obtaining test results in minutes rather than days allows the physician to potentially diagnose the patient during the same visit that the blood was drawn. Patient satisfaction may be increased since there is no need to return for another appointment to discuss lab results and the duration of anxiety while waiting for a diagnosis may be decreased.

Because the goal of the study was to verify the diagnostic performance of the ANA 12 PRI, no impact on the clinical care strategy nor on the socio-economic level have been evaluated. We therefore aim at evaluating the impact of the near patient use of the ANA 12 PRI in routine clinical settings prospectively.

## Supporting information

S1 File RAW DATA. The file contains all the data obtained with the Maverick instruments and in the clincal lab and the corresponding diagnosis.
(XLSX)

## References

[1] American College of Rheumatology Position Statement on Clinical Laboratory Testing; http://www.rheumatology.org/Portals/0/Files/Clinical%20Laboratory%20Testing.pdf.

[2] Konstantinov KN, Tzamaloukas A, Rubin RL (2013) Detection of autoantibodies in a point-of-care rheumatology setting. Auto Immun Highlights 4: 55–61. 10.1007/s13317-013-0052-9 26000143 PMC4389050

[3] Rubin RL, Konstantinov KN (2016) Biosensor for total antinuclear antibody determination at the point-of-care. Biosens Bioelectron 83: 306–311. 10.1016/j.bios.2016.04.048 27132005

[4] Goddard GZ, Soriano A, Gilburd B, Lidar M, Kivity S, Kopilov R, et al (2017) A novel bedside test for ACPA: the CCPoint test is moving the laboratory to the rheumatologist's office. Immunol Res 65: 363–368. 10.1007/s12026-016-8846-2 27470303

[5] Mahler M, Meroni PL, Bossuyt X, Fritzler MJ (2014) Current concepts and future directions for the assessment of autoantibodies to cellular antigens referred to as anti-nuclear antibodies. J Immunol Res 2014: 315179 10.1155/2014/315179 24868563 PMC4020446

[6] Agmon-Levin N, Damoiseaux J, Kallenberg C, Sack U, Witte T, Herold M, et al (2014) International recommendations for the assessment of autoantibodies to cellular antigens referred to as anti-nuclear antibodies. Ann Rheum Dis 73: 17–23. 10.1136/annrheumdis-2013-203863 24126457

[7] Sherer Y, Gorstein A, Fritzler MJ, Shoenfeld Y (2004) Autoantibody explosion in systemic lupus erythematosus: more than 100 different antibodies found in SLE patients. Semin Arthritis Rheum 34: 501–537. 15505768 10.1016/j.semarthrit.2004.07.002

[8] Fritzler MJ (1996) Clinical relevance of autoantibodies in systemic rheumatic diseases. Mol Biol Rep 23: 133–145. 9112221 10.1007/BF00351161

[9] von Muhlen CA, Tan EM (1995) Autoantibodies in the diagnosis of systemic rheumatic diseases. Semin Arthritis Rheum 24: 323–358. 7604300 10.1016/s0049-0172(95)80004-2

[10] Shiboski SC, Shiboski CH, Criswell L, Baer A, Challacombe S, Lanfranchi H, et al (2012) American College of Rheumatology classification criteria for Sjogren's syndrome: a data-driven, expert consensus approach in the Sjogren's International Collaborative Clinical Alliance cohort. Arthritis Care Res (Hoboken) 64: 475–487. 22563590 10.1002/acr.21591 PMC3349440

[11] Petri M, Orbai AM, Alarcon GS, Gordon C, Merrill JT, Fortin PR, et al (2012) Derivation and validation of the Systemic Lupus International Collaborating Clinics classification criteria for systemic lupus erythematosus. Arthritis Rheum 64: 2677–2686. 10.1002/art.34473 22553077 PMC3409311

[12] Amigues JM, Cantagrel A, Abbal M, Mazieres B (1996) Comparative study of 4 diagnosis criteria sets for mixed connective tissue disease in patients with anti-RNP antibodies. Autoimmunity Group of the Hospitals of Toulouse. J Rheumatol 23: 2055–2062. 8970041

[13] van den Hoogen F, Khanna D, Fransen J, Johnson SR, Baron M, Tyndall A, et al (2013) 2013 classification criteria for systemic sclerosis: an American College of Rheumatology/European League against Rheumatism collaborative initiative. Arthritis Rheum 65: 2737–2747. 10.1002/art.38098 24122180 PMC3930146

[14] Willems P, De Langhe E, Claessens J, Westhovens R, Van Hoeyveld E, Poesen K, et al (2018) Screening for connective tissue disease-associated antibodies by automated immunoassay. Clin Chem Lab Med. 10.1515/cclm-2017-0905 29306915

[15] Scholz J, Grossmann K, Knutter I, Hiemann R, Sowa M, Rober N, et al (2015) Second generation analysis of antinuclear antibody (ANA) by combination of screening and confirmatory testing. Clin Chem Lab Med 53: 1991–2002. 10.1515/cclm-2015-0083 26020561

[16] Pasoto SG, Viana VS, Bonfa E (2014) The clinical utility of anti-ribosomal P autoantibodies in systemic lupus erythematosus. Expert Rev Clin Immunol 10: 1493–1503. 10.1586/1744666X.2014.966692 25292164

[17] Pisetsky DS (2016) Anti-DNA antibodies—quintessential biomarkers of SLE. Nat Rev Rheumatol 12: 102–110. 10.1038/nrrheum.2015.151 26581343

[18] Bhansing KJ, Lammens M, Knaapen HK, van Riel PL, van Engelen BG, Vonk MC (2014) Scleroderma-polymyositis overlap syndrome versus idiopathic polymyositis and systemic sclerosis: a descriptive study on clinical features and myopathology. Arthritis Res Ther 16: R111 10.1186/ar4562 24886750 PMC4060195

[19] Dalakas MC, Hohlfeld R (2003) Polymyositis and dermatomyositis. Lancet 362: 971–982. 10.1016/S0140-6736(03)14368-1 14511932

[20] Iqbal M, Gleeson AM, Spaugh B, Tybor F, Gunn W, Hochberg M, et al (2010) Label-Free Biosensor Arrays Based on Silicon Ring Resonators and High-Speed Optical Scanning Instrumentation. IEEE J Sel Quantum Elec 16.

[21] Luchansky MS, Washburn AL, Martin TA, Iqbal M, Gunn LC, Bailey RC (2010) Characterization of the evanescent field profile and bound mass sensitivity of a label-free silicon photonic microring resonator biosensing platform. Biosens Bioelectron 26: 1283–1291. 10.1016/j.bios.2010.07.010 20708399 PMC2997171

[22] Mudumba S, de Alba S, Romero R, Cherwien C, Wu A, Wang J, et al (2017) Photonic ring resonance is a versatile platform for performing multiplex immunoassays in real time. J Immunol Methods. 10.1016/j.jim.2017.05.005 28527901

[23] Werle E, Blazek M, Fiehn W (1992) The clinical significance of measuring different anti-dsDNA antibodies by using the Farr assay, an enzyme immunoassay and a Crithidia luciliae immunofluorescence test. Lupus 1: 369–377. 10.1177/096120339200100606 1304405

[24] Smeenk R, Brinkman K, van den Brink H, Swaak T (1990) A comparison of assays used for the detection of antibodies to DNA. Clin Rheumatol 9: 63–72. 10.1007/BF02205553 2203595

[25] Respaldiza N, Wichmann I, Ocana C, Garcia-Hernandez FJ, Castillo MJ, Magarino MI, et al (2006) Anti-centromere antibodies in patients with systemic lupus erythematosus. Scand J Rheumatol 35: 290–294. 10.1080/03009740600588376 16882593

